# Evolutionary patterns of RNA-based gene duplicates in *Caenorhabditis* nematodes coincide with their genomic features

**DOI:** 10.1186/1756-0500-5-398

**Published:** 2012-08-01

**Authors:** Ming Zou, Guoxiu Wang, Shunping He

**Affiliations:** 1 The key Laboratory of Aquatic Biodiversity and Conservation of Chinese Academy of Sciences, Institute of Hydrobiology, Chinese Academy of Sciences, Wuhan 430072, PR China; 2 University of the Chinese Academy of Sciences, Beijing 100039, PR China; 3 Hubei Key Laboratory of Genetic Regulation and Integrative Biology, HuaZhong Normal University, Wuhan, Hubei, China

**Keywords:** Retrocopy, Chimeric gene, *Caenorhabditis*, Evolutionary pattern

## Abstract

**Background:**

RNA-based gene duplicates (retrocopies) played pivotal roles in many physiological processes. Nowadays, functional retrocopies have been systematically identified in several mammals, fruit flies, plants, zebrafish and other chordates, etc. However, studies about this kind of duplication in *Caenorhabditis* nematodes have not been reported.

**Findings:**

We identified 43, 48, 43, 9, and 42 retrocopies, of which 6, 15, 18, 3, and 13 formed chimeric genes in *C. brenneri*, *C. briggsae*, *C. elegans*, *C. japonica*, and *C. remanei*, respectively. At least 5 chimeric types exist in *Caenorhabditis* species, of which retrocopy recruiting both N and C terminus is the commonest one. Evidences from different analyses demonstrate many retrocopies and almost all chimeric genes may be functional in these species. About half of retrocopies in each species has coordinates in other species, and we suggest that retrocopies in closely related species may be helpful in identifying retrocopies for one certain species.

**Conclusions:**

A number of retrocopies and chimeric genes exist in *Caenorhabditis* genomes, and some of them may be functional. The evolutionary patterns of these genes may correlate with their genomic features, such as the activity of retroelements, the high rate of mutation and deletion rate, and a large proportion of genes subject to trans-splicing.

## Findings

### Background

Gene duplicates that pass through an RNA intermediate termed retrocopies, are a kind of nucleotide sequence formed by the reintegration of retrotranscribed mRNAs into new genomic regions. Retrocopies formed recently have three key features: (1) poly-(A) tail; (2) direct repeats; and (3) lack of intron [1]. Moreover, a retrocopy can incorporate sequences from multiple parental source regions and form a hybrid coding sequence, creating a gene chimera [2,3]. Most retrocopies were thought to be evolutionary dead-ends and functionless, because they probably lacked the expression potential and would be degenerated during evolution [4,5]. However, sporadic studies have found some retrocopies are functional [6-8]. Few years ago, Betran et al. identified numerous retrocopies in *Drosophila* and mammals, and concluded that some of them substituted for their parental genes’s functions to avoid the spermatogenesis X inactivation [9,10]. Subsequently, functional retrocopies have been systematically identified in mammals [11], fruit flies [12], plants [13-15], zebrafish [16] and other chordates [17]. Some retrocopies give evidence of having experienced positive selection [18-22], indicating their potential importance in adaptation.

*C. elegans* is used extensively as a model organism for diverse biological processes. Progress in many research fields, including genetics, molecular biology, and developmental biology, can be attributed to this species. The availability of genome sequences of *C. elegans* and its relatives provide an opportunity for comparative genomics and evolutionary biology to address features of this genus. Generally, the divergence times separating most species of *Caenorhabditis* nematodes span many millions of years [23,24]. Their extent of genome divergence among species can be large, in terms of single nucleotide changes, genome rearrangement, intron gain and loss, and gene family dynamics [23-30]. A large fraction of genes in *Caenorhabditis* genomes can be arranged in operons [31-33] and subject to trans-splicing [34]. Moreover, two species, *C. briggsae* and *C. elegans*, have populations comprised primarily of self-fertilizing hermaphrodites, a mode of reproduction that has originated independently multiple times [28,35]. Many *Caenorhabditis* species are associated with other animals [36]. For example, *C. briggsae* was found in association with snails [37], *C. remanei* was found associated with isopods, snails, and other invertebrates [36], and *C. japonica* was found associated with shield bugs *Parastrachia japonensis* (Heteroptera, Cydnidae) in most of their life time [36,38]. Because of these genomic and organismal features, the evolutionary dynamics of retrocopies that reside in these genomes may be important in adaptive evolution.

Here we identify retrocopies and chimeric genes (together referred to as “new genes” for convenience) in 5 *Caenorhabditis* nematodes: *C. brenneri, C. briggsae, C. elegans,C. japonica,* and *C. remanei* (Additional file 1: Figure S1 shows their phylogenetic relationships). We subsequently compare their abundance among these species. Using expression data from a public databases and RT-PCR experiments, we inferred expression for new genes in *C. elegans*. To explore the functionality of new genes, we deduced open reading frames of retrocopies through comparison to their parental genes in combination with patterns of conservation.

### Methods

#### Datasets compiling and preprocessing

We studied retrocopies in 5 *Caenorhabditis* species (*C. brenneri, C. briggsae, C. elegans, C. japonica,* and *C. remanei*) using available genome sequences. Data (including genome and peptide sequences) were downloaded from WormBase (release WS215) via their ftp server ( ftp://ftp.wormbase.org/). Repetitive sequences in each genome were masked using RepeatMasker [39]. All expression data (ESTs and mRNAs, thereafter called EST for convenience) of *C. elegans* were downloaded from the University of California, Santa Cruz, database (assembly Aug 2010, http://genome.ucsc.edu/). Various contaminants, low quality and low-complexity sequences within these expression data were screened and trimmed using SeqClean [40] with NCBI’s UniVec as a screening file.

#### Retrocopy screening

We modified the retrocopy discovery procedures that have been described elsewhere [41] to identify retrocopies in each genome. Firstly, all peptide sequences were queried against their masked genomic sequences using TBLASTN (E-value ≤ 0.1) [42]. Secondly, a series of customized perl scripts were used to analyze the TBLASTN results and join putative exons together. We selected the best matched DNA segment sequences for each protein and aligned them using GENEWISE [43]. The structure of the selected DNA sequence (exons, introns and whether it is a pseudogene) can be inferred according to its alignment with corresponding protein sequences and we only selected multi-exon sequences for subsequent analyses. On the other hand, we extracted and merged nearby homology matches (distance < 40 bp) that were not likely separated by introns [41] from the TBLASTN results, and required the query and merged target sequences aligned to one another more than 50 amino acids and had amino acid identity more than 30 %. After performing similarity searches of the merged sequences against multi-exon proteins using FASTA [44], we selected the closest hit as their candidate parental protein. To confirm the absence of all introns in retrocopy, we compared the merged sequences with their 10,000-bp flanking regions to their candidate parental proteins using GENEWISE. We also required the GENEWISE score > 35 to ensure they had a certain degree of similarity. At last, we checked the absence of all introns manually and assigned their parental-retrogene relationships for each species.

#### dN and dS estimation and dN/dS ratio test

Pairwise dN and dS statistics for all retrocopies and their parental genes were estimated using the YN00 program of PAML4 [45]. We conducted a likelihood ratio test to determine whether dN/dS between pairs of duplicates was significantly different from 0.5. The Codeml program of PAML4 was run two times (first fixing ω = 0.5 and second estimating omega) for each gene pair and twice of the log likelihood difference of these 2 runs was compared to a χ^2^ distribution with one degree of freedom. dN/dS was smaller than 0.5 and P-value was smaller than 0.01 may denote that the retrocopy is subject to evolutionary constraint and functional [14,41].

#### Identification of Chimerical Retrocopy

Here, we considered a retrocopy as a chimeric retrocopy if the flanking coding sequence(s) that the retrocopy recruits is larger than 50 bp according to the WormBase annotation. If the coding sequence of a protein overlapped more than 90 bp with a retrocopy, we compared the coding sequences that the retrocopy recruits to the genomic sequence and flanking 10,000-bp regions of its parental gene to insure that these recruited sequences derived from other regions rather than parental gene and their flanking regions. We also selected retrocopies that a minimum of two introns from the parental gene were absent in alignable regions that were thought to be definite retrocopies and used clustalx to align them with corresponding chmeric genes to scrutinize their relationships.

#### Distributions of new genes in other *Caenorhabditis* species

To examine the distribution of retrocopies in other *Caenorhabditis* genomes for each species, a procedure similar to screening retrocopies aformentioned was used. Briefly, retrocopies in each species were queried against other 4 *Caenorhabditis* genomes using TBLATX (E-value ≤ 0.1) respectively and nearby homology matches (distance < 40 bp) were extracted and merged. Then, each merged sequence and its flanking 10,000-bp was compared to the corresponding parental protein, and was considered to be a conserved retrocopy in that species when the alignable regions of the query and merged target sequences aligned to one another met the following criteria: (1) longer than 30 amino acids; (2) GENEWISE score > 35; (3) amino acid identity more than 30%; and (4) the merged sequence was intronless. We checked their authenticity manually one by one and discarded false positives. A retrocopy was considered as species-specific if its matched sequences in all other species failed these criteria. For chimeric genes in each species, we identified their othologs in other 4 species using reciprocal best blast hits (RBB) to determine their distribution*.* We checked genomic positions carefully between new genes that identified using different methods to scrutinize their relationships.

#### Pairwise whole genome alignment

Softmasked genome sequences for each species were downloaded from WormBase (release WS215). Pairwise whole genome alignment among *C. brenneri, C. briggsae, C. elegans, C. japonica,* and *C. remanei* was carried out using lastz [46]. Then the Chain/Net package was used for post treatments.

#### Transcription Analysis in *Caenorhabditis* elegans

Here we conducted transcription analysis in *C. elegans* since sufficient expression data in public database for this species were available. To ensure that an EST is derived from a retrocopy rather than its parental gene, we followed a relatively complicated pipeline [14] to retain high-quality mappings. We mapped all 391,185 cleaned sequences to genome sequences using BLAT [47], and retained 280241 sequences that meet the following criteria: mapping length ≥ 150 bp, identity ≥ 98%, coverage within mapping ≥ 97%, and coverage within whole transcript ≥ 75%. We selected the best mapping if a transcript was mapping to multiple genomic loci and discarded those ambiguous mappings (difference in BLAT scores < 2%). Finally, we obtained high-quality and clearly mappings for 277,204 ESTs (71%). Subsequently, we compared genomic positions of retrocopies and the mapped positions of ESTs. A retrocopy was doubtless expressed if it overlapped more than 150 bp with an EST, and probably expressed if it overlapped more than 100 bp with an EST (including ambiguious mappings). For chimeric gene, we mapped its coding sequence to cleaned expression data using BLASTN (E-value ≤ 1e-20), and considered it was expressed if the mapped EST contained sequences longer than 50 bp both from retrocopy and recruited sequences. Only 2 chimeric genes failed this criterion. Expression of new genes lacking EST supports was checked using RT-PCR experiments.

#### RT-PCR experiments

Total RNA was extracted from *C elegans* samples (N2 strain, mixed stage) using trizol reagent (Invitrogen, Carlsbad, CA, USA). To avoid genomic contamination, we treated Total RNA with DNase I (Promega) to remove genomic DNA. Unique primers were used to amplify target sequences, and mRNAs without being reverse-transcribed was used as negative controls. Purified products were sequenced on an ABI 3730 DNA Analyzer (Applied Biosystems, Foster City, California, United States) and the resulting sequences were deposited in GenBank [GenBank: JN655873-JN655883].

### Results and discussions

#### Retrocopies in *Caenorhabditis* nematodes

Using a modified retrocopy discovery procedure [41], we identified retrocopies in 5 *Caenorhabditis* genomes (*C. brenneri, C. briggsae, C. elegans, C. japonica,* and *C. remanei*), as summarized in Table 1 (for more information, please see Additional file 2: Table S1). At first, we obtained 141, 90, 82, 365, 134 retrocopies for *C. brenneri, C. briggsae, C. elegans, C. japonica,* and *C. remanei*, respectively. However, these numbers may be overestimated because of the following reasons: Firstly, in *C. brenneri, C. japonica,* and *C. remanei*, the genome assembly is fragmented and containing many short scaffolds, this may result in false positives owing of a potential failure to detect some introns. Secondly, the nematode introns are lost at a very high rate, thus some false positives may be paralogues that lost introns during evolution [27]. Thirdly, natural heterozygosity may cause part of genome assemblies represented by alleles, especially for gonochoristic *Caenorhabditis* species [48]. To overcome these problems, we required retrocopies reside in chromosomes (or scaffolds) longer than 50 kb, and in different chromosomes (or scaffolds) compared with their parental genes. If multiple retrocopies share one parental gene, we selected the one located in the longest chromosome, or have lowest dS values compared to the parental gene. As a result, we obtained 43, 48, 43, 9, 42 retrocopies for *C. brenneri, C. briggsae, C. elegans, C. japonica,* and *C. remanei*, respectively (Table 1).

**Table 1:** **Summaries of retrocopies reside in the five *Caenorhabditis *nematodes**

**Species**	**N(r)**	**N(i)**	**N(f)**	**N(s)**	**N(f & s)**	**N (0.5)**
*C. brenneri*	43	28	12	1	2	29
*C. briggsae*	48	35	11	0	2	36
*C. elegans*	43	37	5	1	0	32
*C. japonica*	9	4	3	0	2	3
*C. remanei*	42	24	11	2	5	23

N (r): Number of retrocopies; N(i): Number of intact retrocopies; N(f): Number of frameshift retrocopies; N(s): Number of retrocopies have premature stop codons; N(f & s): Number of retrocopies both have frameshifts and premature stop codons; N (0.5): Number of retrocopies have omega values (between them and corresponding parental genes) less than 0.5 significantly.

A retrocopy is thought to be functional if it meets the following criteria: its open reading frame is intact; it is subjected to purifying selection; and it is expressed. Here, we inferred the open reading frames of retrocopies by comparing alignment regions with their parental genes. To explore the conservation of retrocopies, we examined the ratio of nonsynonymous substitutions per site (dN) and synonymous substitutions per site (dS) between them and their functional parental genes. dN/dS (also terme ω) significantly lower than 0.5 indicate functional constraint on both genes [9]. Consequently, we determined that the open reading frames of majority of retrocopies in each species were intact (Table 1, and Additional file 2: Table S1), and that a small number of them were disrupted (by frameshift mutations or premature stop codons, or both). We also found most retrocopies were subject to purifying selection in *Caenorhabditis* nematodes (Table 1, Additional file 2: Table S1). We obtained expression profiles for retrocopies in *C. elegans* using expression data available from a public database. Out of 43 retrocopies in total, 33 were confidently determined to be expressed, and 1 were probably expressed (Additional file 2: Table S1). Additionally, we confirmed the expression for eight other retrocopies via RT-PCR experiments, and found the open reading-frame of the only retrocopy do not have any expression support was disabled. In summary, in *C. elegans*, all 32 retrocopies subjected to purifying selection are expressed, and 5 disrupted retrocopies are also expressed (Additional file 2: Table S1).

Taken together, majority of retrocopies have intact open reading frames and are subjected to purifying selection in *Caenorhabditis* nematodes, although some others possess interrupted open reading frames are still subject to purifying selection (5, 5, 3, 7 retrocopies in *C. brenneri, C. briggsae, C. japonica,* and *C. remanei*, respectively). In *C.elegans*, only one retrocopy with interrupted open reading frames were not supported by expression data. Therefore, we suggest that a substantial portion of retrocopies in the genomes of *Caenorhabditis* species are likely functional. This pattern is similar to that in non-mammal chordates [17]. This might result from loss of most non-functional retrocopies owing to the high mutation and deletion rate in *Caenorhabditis*[49,50], with only the small fraction of functional retrocopies retained over evolutionary time.

#### Chimeric genes in *Caenorhabditis* nematodes

**Figure 1:**
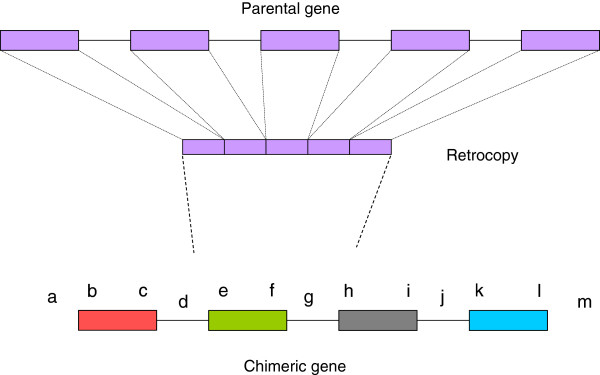
**All chimeric types exist in *Caenorhabditis *nematodes (Boxes and solid lines represent exons and introns, respectively).** I: retrocopy becomes (part of) the first exon in chimeric gene (region bc); II: retrocopy becomes (part of) the last exon in chimeric gene (region kl); III: retrocopy becomes (part of) the exon between the first and the last one (region ef); IV: retrocopy becomes the first exon and part of 5’ non-coding region in chimeric gene (region ac); V: retrocopy becomes 2 exons and the intron between them (region ei).

We found 6, 15, 18, 3, 13 retrocopies recruited nearby regions and formed chimeric genes in *C. brenneri, C. briggsae, C. elegans, C. japonica,* and *C. remanei*, respectively. Table 2 summarizes attributes of chimeric genes in these 5 *Caenorhabditis* species (more information in Additional file 2: Table S1). The open reading frames of a large number of chimeric retrocopies are intact compared to their parental genes (Table 2). However, there are still some chimeric retrocopies have interrupted open reading frames. To figure out how interrupted retrocopies form chimeric genes, we scrutinize the structures of retrocopies and corresponding chimeric genes. This analyses was performed for retrocopies that lost at least 2 parental introns to ensure they truly originated by retroposition and the results are shown in Table 2 (please see Additional file 2: Table S1 for details). At least 5 chimeric types exist in *Caenorhabditis* species with more types in *C. elegans* and *C. remanei* (Figure 1). In accordance with results in zebrafish, type III chimeric genesare the commonest in *Caenorhabditis* species [16]. That is to say, about three-quarter of chimeric genes subjected to our analysis recruited both N and C terminus and formed new chimeric coding sequences, and others are comprised of all kinds of other chimeric types. It is interesting to note that frameshift mutations or premature stopcodons always located in the noncoding regions of chimeric gene, such as upstream of the start codon and introns (Figure 1). The phenomenon that part of retrocopy sequence transformed into non-coding sequences in chimeric genes has been reported before in other species [14,16], indicating its generality. These results suggested that both intact and interrupted retrocopies can form chimeric genes and the interrupted regions transformed into non-coding regions in chimeric genes, no matter what chimeric types they are.

**Table 2:** **Summaries of chimeric retrocopies reside in the five *Caenorhabditis *nematodes**

**Species**	**N (C)**	**N (i)**	**N (f)**	**N (s)**	**N (f & s)**	**N (0.5)**	**Chimeric type(s)**
*C. brenneri*	6	5	1	0	0	5	III (1)
*C. briggsae*	15	14	0	0	1	13	III (7)
*C. elegans*	18	17	1	0	0	14	I (1), III (2), IV (1)
*C. japonica*	3	2	1	0	0	2	--
*C. remanei*	13	8	4	0	1	10	II (1), III (2), V(1)

N (C): Number of chimeric retrocopies; N (i): Number of intact chimeric retrocopies; N (f): Number of frameshift chimeric retrocopies; N (s): Number of chimeric retrocopies have premature stop codons; N (f & s): Number of chimeric retrocopies both have frameshifts and premature stop codons; N (0.5): Number of chimeric retrocopies have omega values (between them and corresponding parental genes) less than 0.5 significantly; chimeric type(s): the chimeric type are represented in roman numbers (Figure 1 for more information), inside brackets are number of chimeric type.

Table 2 also shows the ωvalues for many retro-parental gene pairs are significantly less than 0.5. Omega values less than 0.5 was a conservative denotation that both parental gene and retrocopy are subjected to purifying selection [9]. Therefore, some cases that the ω values less than 0.5 significantly may be true, but some others may be false positives and more robust tests should be used since newly originated genes are subject to weaker purifying selection or positive selection frequently during their evolution [51]. We confirmed the expression support by either mRNA or EST sequences in the public databases for 17 out of 18 chimeric genes in *C. elegans* (Additional file 2: Table S1)*.* However, we failed to testify the expression for the other one chimeric retrocopies using RT-PCR experiments. The results that many chimeric retrocopies are under evolutionary constraints and are expressed, which indicated majority of chimeric genes in *Caenorhabditis* species may be functional. It is interesting that many functional chimeric genes reside in the small *Caenorhabditis* genomes. A possible reason may be that majority of transcripts in these species are subject to trans-splicing [52,53]. These trans-spliced transcripts could be retro-transcribed and integrated into new genomic regions and formed chimeric genes [2]. *Caenorhabditis* nematodes are relatively constant, thus some of these newly originated chimeric genes are retained and modified adaptively to accommodate the environmental change.

#### Distributions of retrocopy and chimeric genes

We screened each species of *Caenorhabditis* for homologous copies of new genes in the other four species (see methods), with their distribution shown in Additional file 3: Table S2 and Additional file 4: Table S3. In *C. brenneri, C. briggsae, C. elegans, C. japonica,* and *C. remanei*, we found 22, 13, 13, 5, 18 retrocopies and 2, 3, 3, 1, 5 chimeric genes are species-specific. This number may be underestimated for retrocopies since the high lost rate of introns in *Caenorhabditis* nematodes [27]. As a result, we take into account of results from whole genome alignment (synteny), and found 19, 25, 22, 3, 18 retrocopies in *C. brenneri, C. briggsae, C. elegans, C. japonica,* and *C. remanei* have coordinates in other species (Additional file 3: Table S2), and the analyses following are based on these retrocopies. In a previous research, Bai et al. found that only 1 retrogene in the *D. melanogaster* genome was species-specific [12]. Our research demonstrated that more retrocopies were species-specific and less was common in genus *Caenorhabditis*. These results corroborate the high divergence of *Caenorhabditis* nematodes, which have been reported in previous studies [28,54].

Generally, the dS values of retrocopies have coordinates in other *Caenorhabditis* species are higher than those retrocopies that are species-specific (P = 0.053, 0.016, 0.057, 0.007 in *C. brenneri, C. briggsae, C. elegans,* and *C. remanei*, Mann–Whitney U-test). The differences should be significant statistically given that nonparametric tests have less "power" to detect a significant difference. The majority of retrocopies have coordinates in other *Caenorhabditis* species are intact compared to the open reading frame of their parental gene. The same is true for the proportion of retrocopies whose ω values significantly less than 0.5. On the contrary, more than half of species-specific retrocopies have frameshift mutations or premature stop codons compared to their parental sequences except in *C. elegans*, the proportion of which was as much as 30.8 %. Only a small portion of species-specific retrocopies in each species have omega values significantly less than 0.5. In summary, retrocopies have coordinates in other *Caenorhabditis* species are older than species-specific ones, and their retention indicated that they have essential functions and are under evolutionary constrains. However, since new genes originated recently can quickly become essential [55,56], some of these species-specific retrogenes should have functions in *Caenorhabditis* and will be good candidates for subsequent functional experiments.

We compared chromosome positions of retrocopies identified using different methods, and found only a small portion of them overlapped (data not shown). The number of overlapped retrocopies seems to correlate with their phylogenetic positions since more overlapped retrocopies were found using retrocopies of closely related species as queries and less overlapped were found using retrocopies of distantly related species. We obtained the same distribution pattern for chimeric genes. This may be due to that the mutation rate in *Caenorhabditis* species is high [28,50,57], plus the deletion rate of neutral regions in *Caenorhabditis* genomes is fast [49,50]. As a result, retrocopies in these species degenerate fast if they do not function immediately. On the other hand, the sequencing, assembly and annotations for some genomes may be incomplete or have some problems, or other reasons resulted in the absence of parental genes formed the obstacle for obtaining full list of retrocopies in these species. However, we could find out retrocopies that have not been found by just screening one genome, using retrocopies of closely related species to perform homologous screening. We suggest this method should be complementary and useful for identifying retrocopies in one species when the retrocopies of its closely related species can be available. The high mutation rate in *Caenorhabditis* species may also explain the phenomenon that chimeric genes formed by species-specific retrocopies are not necessarily species-specific.

### Conclusions

Here, we identified and compared retrocopies in 5 *Caenorhabditis* nematodes and explored their functionality. Most retrocopies have intact open reading frames and are conservative suggesting that a majority of retrocopies in each genome may be functional. Moreover, the expression data from public database and RT-PCR experiments demonstrated almost all retrocopies in *C. elegans* are expressed. In *Caenorhabditis* nematodes, at least 5 chimeric types exist,and the most common type is retrocopy recruiting both N and C terminus and forming new chimeric coding sequences. Interrupted retrocopies can form chimeric genes in these species,and the interrupted region transformed into non-coding regions. Using homology screening, we obtained the distribution in other 4 *Caenorhabditis* species for each retrocopy and chimeric gene.

## Competing interests

All authors declare that they have no competing interests.

## Authors’ contributions

MZ carried out the analyses and drafted the manuscript. SH designed and participated in the analyses. GW participated in the design and helped to draft the manuscript. All authors read and approved the final manuscript.

## Supplementary Material

Additional file 1 **Figure S1.** The phylogeny of the 5 *Caenorhabditis* nematodes subjected to analyses in this study (adopted from Kiontke et al. [35]). Click here for file

Additional file 2 **Table S1.** Summary of retrocopies and chimeric genes identified in 5 *Caenorhabditis* nematodes. Click here for file

Additional file 3 **Table S2.** Distribution of retrocopies in other 4 *Caenorhabditis* nematodes. Click here for file

Additional file 4 **Table S3.** Distribution of chimeric genes in other 4 *Caenorhabditis* nematodes. Click here for file
