# Supplementary figures and images for: Evolutionary patterns of RNA-based gene duplicates in Caenorhabditis nematodes coincide with their genomic features
Source: BMC Res Notes. 2012 Aug 1;5:398. doi: 10.1186/1756-0500-5-398 (PMC3532220; doi:10.1186/1756-0500-5-398)

## Slide 1
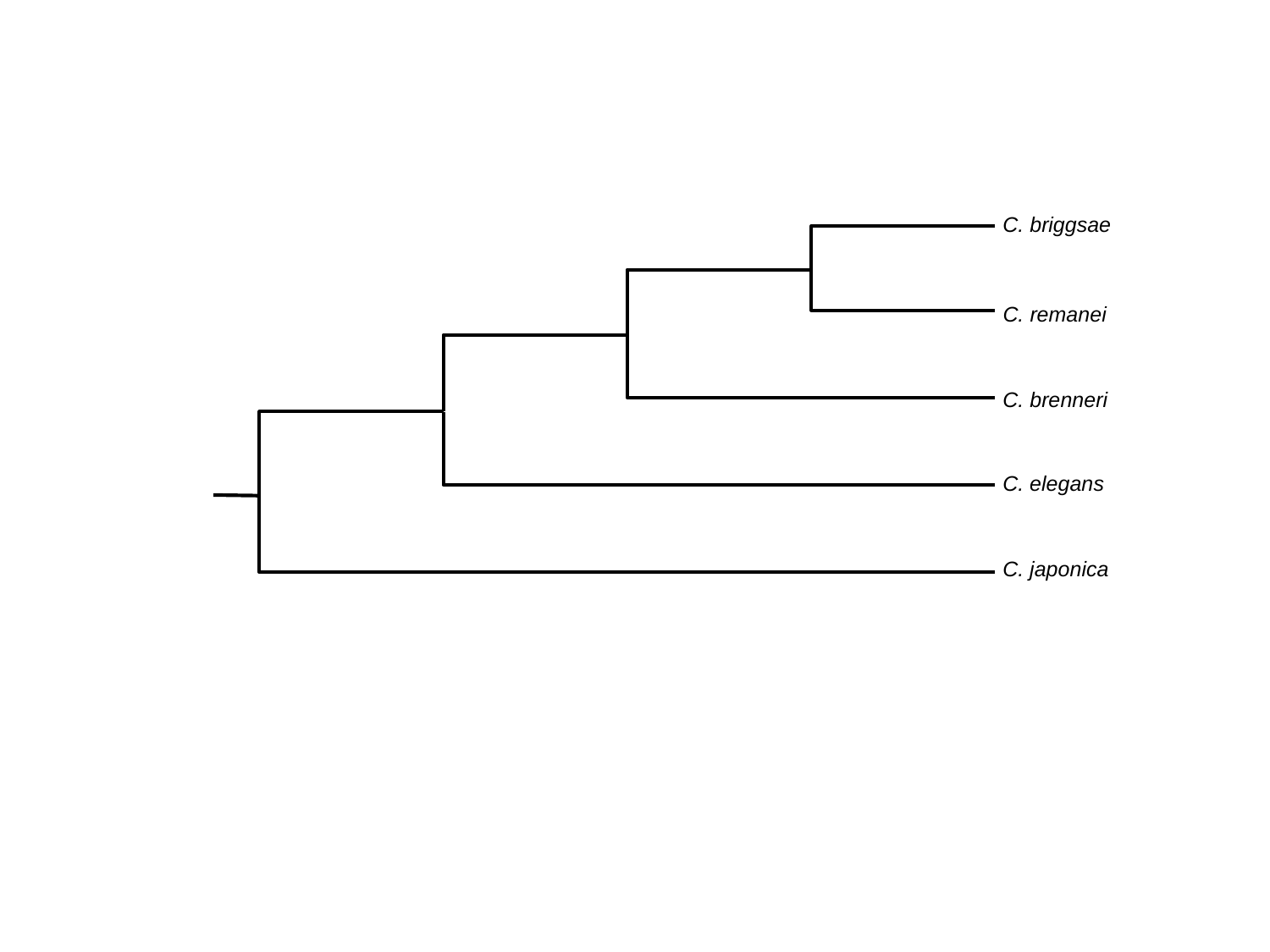

C. briggsae
 C. remanei
 C. brenneri
 C. elegans
 C. japonica

Supplement: Additional file 1 — Figure S1. The phylogeny of the 5 Caenorhabditis nematodes subjected to analyses in this study (adopted from Kiontke et al. [35]). [file 1756-0500-5-398-S1.ppt]
